# Retraction: High performance liquid chromatography coupled with high resolution mass spectrometry-based characterization of lipidomic responses from rats with kidney injuries

**DOI:** 10.1039/d1ra90054d

**Published:** 2021-02-03

**Authors:** Laura Fisher

**Affiliations:** Royal Society of Chemistry Thomas Graham House, Science Park, Milton Road Cambridge CB4 0WF UK advances-rsc@rsc.org

## Abstract

Retraction of ‘High performance liquid chromatography coupled with high resolution mass spectrometry-based characterization of lipidomic responses from rats with kidney injuries’ by Qun Liang *et al.*, *RSC Adv.*, 2018, **8**, 20250–20258, DOI: 10.1039/C8RA02805B.

The Royal Society of Chemistry hereby wholly retracts this *RSC Advances* article due to concerns with the reliability of the data. The images in the article were screened by an image integrity expert. Images published in the article have also been published in two other papers, one by the same authors^1^ but another by a different set of authors.^2^

The left and centre panels of Fig. 2A represent different sections of the same image. Furthermore, the same image is also duplicated in ref. 1 but representing different experiments.

The right panel in Fig. 2A and the left panel in Fig. 2B are identical to images published in ref. 2, which has no overlapping authors to this *RSC Advances* article.

Given the significance of the concerns about the validity of the data, the findings presented in this paper are not reliable.

Qun Liang does not agree with this retraction. The other authors were informed but have not responded to any correspondence regarding the retraction.

Signed: Laura Fisher, Executive Editor, *RSC Advances*

Date: 19^th^ January 2021

## Supplementary Material
